# Critical role of CRAG, a splicing variant of centaurin-γ3/AGAP3, in ELK1-dependent SRF activation at PML bodies

**DOI:** 10.1038/s41598-019-56559-9

**Published:** 2019-12-27

**Authors:** Shun Nagashima, Keisuke Takeda, Isshin Shiiba, Mizuho Higashi, Toshifumi Fukuda, Takeshi Tokuyama, Nobuko Matsushita, Seiichi Nagano, Toshiyuki Araki, Mari Kaneko, Go Shioi, Ryoko Inatome, Shigeru Yanagi

**Affiliations:** 1 Laboratory of Molecular Biochemistry, School of Life Sciences, Tokyo University of Pharmacy and Life Sciences, Hachioji, Tokyo 192-0392 Japan; 2 Department of Peripheral Nervous System Research National Institute of Neuroscience, National Center of Neurology and Psychiatry, Kodaira, Tokyo Japan; 3 Animal Resource Development Unit, RIKEN Center for Life Science Technologies, Kobe, Japan; 4 Genetic Engineering Team, Division of Bio-function Dynamics Imaging, RIKEN Center for Life Science Technologies, Kobe, Japan

**Keywords:** Cell growth, Cell death in the nervous system

## Abstract

CRMP-5-associated GTPase (CRAG), a short splicing variant of centaurin-γ3/AGAP3, is predominantly expressed in the developing brain. We previously demonstrated that CRAG, but not centaurin-γ3, translocates to the nucleus and activates the serum response factor (SRF)-c-Fos pathway in cultured neuronal cells. However, the physiological relevance of CRAG *in vivo* is unknown. Here, we found that CRAG/centaurin-γ3–knockout mice showed intensively suppressed kainic acid-induced c-fos expression in the hippocampus. Analyses of molecular mechanisms underlying CRAG-mediated SRF activation revealed that CRAG has an essential role in GTPase activity, interacts with ELK1 (a co-activator of SRF), and activates SRF in an ELK1-dependent manner. Furthermore, CRAG and ELK1 interact with promyelocytic leukaemia bodies through SUMO-interacting motifs, which is required for SRF activation. These results suggest that CRAG plays a critical role in ELK1-dependent SRF-c-fos activation at promyelocytic leukaemia bodies in the developing brain.

## Introduction

By screening for signalling targets of repulsive axon guidance factors, semaphorins, we previously identified CRAM (CRMP-5)-Associated GTPase (CRAG), a neuron-specific guanosine triphosphatase (GTPase) that is an alternative splicing variant of centaurin-γ3/AGAP3^1^. CRAG contains a nuclear localization signal (NLS) at the C-terminus that is required for nuclear translocation in response to various stimuli including semaphorin 3A or reactive oxygen species production^2^. Therefore, CRAG might be involved in redox signalling in neurons. However, its function has yet to be clarified. We previously found that CRAG facilitates the degradation of expanded polyglutamine protein (polyQ) via the nuclear ubiquitin-proteasome pathway^1^. Taking advantage of this feature, we also showed that lentivirus-mediated CRAG expression in the Purkinje cells of mice expressing polyQ resulted in clearance of the polyQ aggregates and rescued ataxia^3^. These results suggest that targeted delivery of CRAG might be useful as a gene therapy for neurodegenerative diseases such as polyglutamine diseases^3^. Thus, CRAG might protect neurons against the accumulation of misfolded proteins by activating the nuclear ubiquitin proteasome system.

In addition, we previously reported that CRAG, but not centaurin-γ3, induces the transcriptional activation of c-Fos–dependent activator protein-1 (AP-1) via serum response factor (SRF) in the nucleus^4^. We showed that CRAG knockdown by siRNA or expression of a dominant-negative mutant of CRAG in cultured neuronal cells significantly reduces the c-Fos activation triggered by polyQ accumulation. In addition, CRAG might be involved in the sulfiredoxin-mediated antioxidant pathway via AP-1^4^. Therefore, CRAG might protect neuronal cells from the accumulation of unfolded proteins, not only through proteasome activation, but also through SRF-mediated c-Fos activation. However, the molecular mechanism underlying CRAG-induced SRF activation remains entirely unknown. In the present study, we analyzed CRAG/centaurin-γ3–knockout (KO) mice and found that CRAG plays a critical role in ELK1-mediated SRF activation. Furthermore, we found that promyelocytic leukaemia bodies are important in CRAG-induced SRF activation.

## Results

### Kainic acid-induced c-Fos expression is attenuated in the hippocampus in CRAG-KO mice

We previously demonstrated that CRAG induces SRF-c-Fos activation in cultured neuronal cells^4^. However, the physiological significance of CRAG in SRF-c-Fos activation is unknown. To investigate whether CRAG activates SRF *in vivo*, we generated CRAG/centaurin-γ3–KO mice. Several splicing variants of centaurin-γ3/AGAP3 are identified in the NCBI database (Gene ID: 116988), including CRAG and a GTP domain-deleted isoform (Supplementary Fig. S1A). The GTPase domain is not required for centaurin-γ3–mediated, Arf6-mediated vesicle transport^5^, suggesting that the GTP domain has an independent and intrinsic function distinct from that of other centaurin-common regions containing the pleckstrin homology (PH), Arf Gap, and ankyrin repeat domains. CRAG/centaurin-γ3 whole-body KO (WKO) mice were established by deleting the GTPase domain (Supplementary Fig. S1B–F). A short GTPase domain-deleted variant of centaurin-γ3–containing centaurin-common regions was upregulated in the brains of these mutant mice (Supplementary Fig. S1F), suggesting that this variant compensates for the loss of function of the centaurin-common domains that occurs in centaurin-γ3. These mutant mice specifically lost the CRAG and GTPase domain functions of centaurin-γ3. In contrast to wild-type (WT) CRAG, neither the CRAG mutant lacking the GTPase domain nor the full length centaurin gamma-3 was capable of activating an SRF reporter gene (Supplementary Fig. S1G), these mutant mice provide a direct means of determining the physiological role of CRAG in SRF activation *in vivo*.

**Figure 1:**
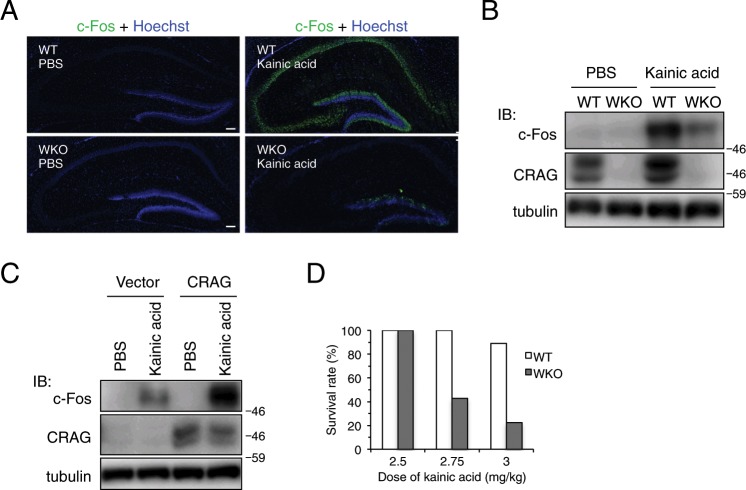
CRAG is partially required for kainic acid-induced c-Fos expression in the mouse hippocampus. (**A,B**) Comparison of c-Fos expression in the hippocampus between WT and WKO mice at P18. c-Fos expressions were detected by immunostaining (**A**) and immunoblotting (**B**) in the hippocampus at 3 hours following PBS or 3 mg/kg kainic acid injection. Scale bars represent 100 μm. (**C**) Lentiviral expression of CRAG rescued kainic acid-induced c-Fos in primary cultured hippocampal neurons derived from WKO mice. After 1 day *in vitro*, cells were infected by lentiviral expression vector with CRAG. Lysates of primary hippocampal neurons of WKO mice at 3 hours following PBS or 200 μM kainic acid stimulation at 9 days *in vitro* were immunoblotted with indicated antibodies. (**D**) WKO mice exhibited a high fatality rate even after administration of low concentrations of kainic acid at P18-24. (*n* = 3 for 2.5 mg/kg kainic acid injection, *n* = 7 for 2.75 mg/kg kainic acid injection, and *n* = 9 for 3 mg/kg kainic acid injection).

A luciferase assay in Neuro2A cells revealed that CRAG, but not centaurin-γ3, activated the *c-fos* promoter (Supplementary Fig. 1H), corroborating our previous observations. Dominant-negative SRF Δ338 mutant inhibited CRAG-induced SRF activation, indicating that CRAG activates *c-fos* through SRF activation. To understand the role of CRAG in c-Fos induction *in vivo*, we treated WKO mice with kainic acid, an agonist of the kainate-class ionotropic glutamate receptor and a strong inducer of c-Fos expression^6,7^. We tested whether CRAG was required for c-Fos expression after kainic acid treatment. Upon phosphate-buffered saline (PBS) treatment as a control, c-Fos expression was undetectable in both the WT and WKO hippocampi (Fig. 1A, left). Kainic acid treatment rapidly induced c-Fos production in the WT hippocampi, whereas c-Fos induction was severely attenuated in the WKO hippocampi (Fig. 1A, right). Consistently, immunoblot analysis indicated that kainic acid-induced c-Fos expression was strongly suppressed in the WKO hippocampi compared to that in the WT hippocampi (Fig. 1B). Moreover, lentiviral vector-mediated CRAG expression rescued c-Fos induction in kainic acid-stimulated primary cultured WKO hippocampal neurons (Fig. 1C). There was no significant difference in SRF expression between WT and WKO hippocampi, indicating that CRAG KO did not affect SRF expression (Supplementary Fig. S1F). WKO mice had high death rates upon kainic acid treatment (Fig. 1D), which is consistent with the previous finding that both SRF-KO and *c-fos*–KO mice are vulnerable to kainic acid treatment^6,8^. Therefore, these results suggest that CRAG is required for c-Fos induction by kainic acid stimulation *in vivo*.

### CRAG activates SRF through interaction with ELK1

**Figure 2:**
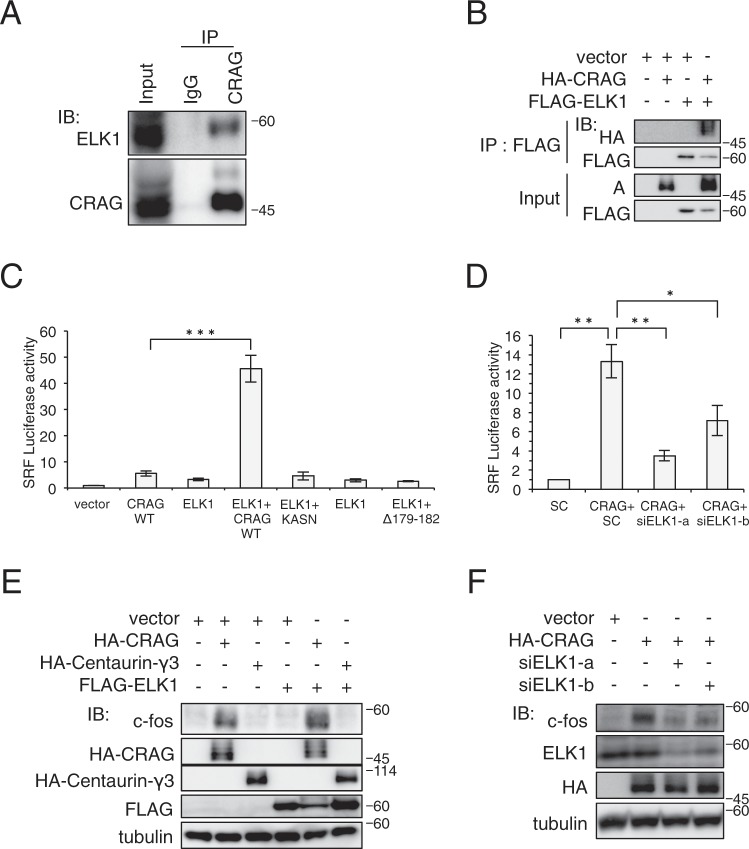
CRAG activates SRF in an ELK1-dependent manner. (**A**) Interaction of endogenous CRAG with ELK1 in mouse brain. Lysates from mouse brain were subjected to IP with anti-CRAG antibody or normal rabbit IgG followed by IB with indicated antibodies. (**B**) Interaction of HA-CRAG with FLAG-ELK1 in cell expression system. Lysates of Neuro2a cells transfected with the indicated vectors were sonicated and subjected to an IP-IB assay with the indicated antibodies. These blots of HA and FLAG were obtained from different exposure times between IP: FLAG and Input depending on signal intensities. (**C**) Synergistic activation of SRF by CRAG and ELK1. (**D**) ELK1 knockdown attenuated CRAG-induced SRF activation. (**C,D**) Luciferase assay was performed with Neuro2A cells transfected with both pSRF-Luc and pRL-CMV with indicated vector and/or siRNA (*sc*: scramble siRNA, *siELK1-a and siELK1-b*: ELK1-specific siRNA). (*n* = 3; *P < 0.05, **P < 0.01, ***P < 0.005, *t-*test). Error bars indicate S.D. (**E**) Enhanced induction of c-Fos by co-expression of CRAG and ELK1. (**F**) ELK1 knockdown reduced c-Fos induction by CRAG. Lysates of Neuro2A cells were immunoblotted with the indicated antibodies (**E,F**).

To gain insight into the molecular mechanism of CRAG-mediated SRF activation, we focused on ELK1, a cofactor of SRF^9,10^. Accordingly, we found that CRAG interacts with ELK1; an immunoprecipitation assay demonstrated that endogenous ELK1 was co-precipitated with endogenous CRAG in the mouse brain (Fig. 2A). To confirm that CRAG directly interacts with ELK1, we performed an immunoprecipitation assay on Neuro2a cells transfected with tagged CRAG and ELK1 expression vectors. CRAG was co-precipitated with FLAG-ELK1 (Fig. 2B) but not Myc-SRF (Supplementary Fig. S2A). A luciferase reporter assay further revealed that co-expression of CRAG with ELK1 dramatically increased SRF activation (by more than 40 fold) (Fig. 2C), suggesting that a close functional link between CRAG and ELK1. Consistently, ELK1 knockdown by siRNA reduced SRF activation and c-Fos induction by CRAG (Fig. 2D,F). Immunoblot analysis revealed slightly enhanced induction of c-Fos by CRAG and ELK1 co-expression (Figs. 2E and S2B), although the synergistic effect was much smaller than that in the SRF-driven luciferase reporter assay using pSRF-Luc. Similarly, a luciferase assay using *c-fos* promoter revealed *c-fos* activation of (although less than 2 times that with CRAG alone) by co-expression of CRAG and ELK1 (Supplementary Fig. S2C). Therefore, c-Fos induction likely becomes saturated upon SRF activation by the overexpression of CRAG alone. Taken together, these findings indicate that CRAG activates SRF–c-Fos through ELK1. Various CRAG mutants, which were unable to translocate to the nucleus, failed to synergistically activate SRF with ELK1 (Supplementary Fig. S2D). To confirm the ELK1-dependent SRF activation by CRAG, we examined the effect of the ELK1 ΔETS mutant, which lacks transcriptional activity for SRF, on CRAG-induced SRF activation. As expected, ELK1 ΔETS inhibited SRF activation induced by CRAG (Supplementary Fig. S2E). Next, we examined the effects of other ELK-family members on CRAG-mediated SRF activation: CRAG synergistically activated SRF with ELK4 but not with ELK3 (Supplementary Fig. S2F). ELK1 has been shown to be activated through phosphorylation by ERK, JNK, or p38^9^. Accordingly, the MEK inhibitor U0126 partially blocked CRAG- and ELK1-induced SRF activation (Supplementary Fig. S2G), suggesting that CRAG activates ELK1, at least in part, by phosphorylation through the MEK pathway. However, the non-phosphorylated ELK1 mutant S384/390A only slightly inhibited CRAG-mediated SRF activity (Supplementary Fig. S2H). Therefore, these results indicate that both ELK1-dependent and -independent mechanisms are involved in the CRAG-mediated SRF activation.

### CRAG GTPase activity is critical for CRAG-induced SRF activation

**Figure 3:**
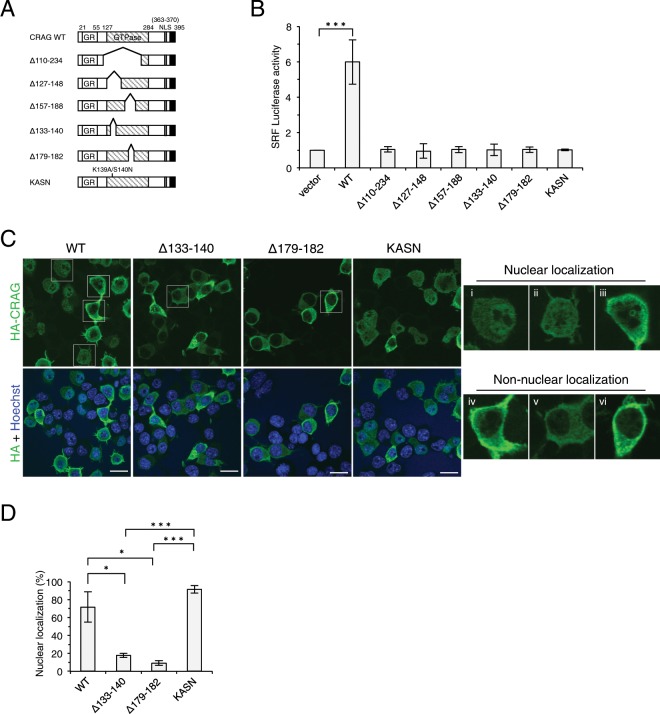
GTPase in CRAG is critical to activate SRF. (**A**) Structural comparison of CRAG GTPase mutants. 133–140 amino acids in CRAG are phosphate-binding motif. 179–182 amino acids in CRAG are Mg^2+^-binding motif. *GR*, Glycine rich domain; *NLS*, nuclear localization signal. Filled black indicates CRAG-specific C-terminal domain. (**B**) CRAG GTPase mutants fail to activate SRF. Neuro2A cells were transfected with both pSRF-Luc and pRL-CMV with indicated vector. Luciferase activities were assessed 24 hours after the transfection. (*n = *4; ***P < 0.005, *t*-test). (**C**) Subcellular localizations of CRAG GTPase mutants. Neuro2A cells were transfected with either HA-CRAG-WT, Δ133–140, Δ179–182 or KASN. Cells were immunostained with anti-HA (green) and Hoechst 33258 (blue) 24 hours after the transfection. The panels on the right show high-magnification images of the boxed regions. CRAG localization patterns were classified as follows: (i) nuclear rich localization, (ii) nuclear and cytoplasmic localization, (iii) nuclear aggregation, (iv) plasma membrane-rich localization, (v) cytoplasmic localization, and (vi) cytoplasmic aggregation. Scale bars represent 20 µm. (**D**) The percentage of cells showing nuclear localization of CRAG and GTPase mutants. (*n = *3 independent experiments, quantifying at least 50 cells from three coverslips within each experiment; *P < 0.05, ***P < 0.005, *t*-test). All error bars indicate S.D.

To explore the mechanism of CRAG-induced SRF activation, we focused on the role of the CRAG GTPase domain. Previous studies show that CRAG GTPase mutant (S140N) exhibits nuclear localization and AP-1 activation^1,4^. We generated new CRAG GTPase mutants to test whether the CRAG GTPase domain is required for SRF activation (Fig. 3A). A luciferase assay demonstrated that WT CRAG GTPase activated SRF, whereas cells expressing CRAG GTPase mutants including CRAG K139A/S140N mutant (KASN) showed no significant SRF activation (Figs. 3B and S3A). We checked the GTP-binding ability of the KASN mutant using GTP-agarose. In contrast to WT CRAG, the KASN mutant failed to bind GTP (Supplementary Fig. S3B,C). Moreover, CRAG GTPase mutants had no synergistic effect on ELK1-mediated SRF activation. Thus, GTPase activity in CRAG is critical for SRF activation through ELK1 (Fig. 2C). Furthermore, unlike CRAG-WT, which was localized to the nucleus in approximately 70% of the cells, GTPase-deleted CRAG mutants failed to translocate to the nucleus (Fig. 3C). On the other hand, KASN predominantly localized to the nucleus (Fig. 3D), indicating that the CRAG GTPase domain is essential for SRF activation in the nucleus. Thus, the CRAG GTPase domain regulates CRAG function.

### CRAG activates SRF by interacting with PML through its SUMO-interacting motif domains

**Figure 4:**
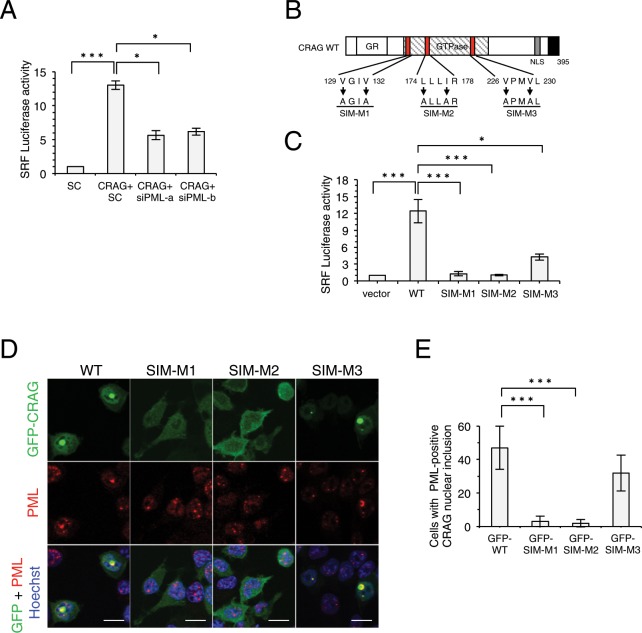
CRAG activates SRF via interaction with PML through SIM domains. (**A**) PML knockdown attenuated CRAG-induced SRF activation. Neuro2A cells transfected with both pSRF-Luc and pRL-CMV with indicated vector and/or siRNA (sc: scramble siRNA, siPML-a and siPML-b: PML-specific siRNA). Luciferase activities were assessed 48 hours after the transfection (*n* = 3; *P < 0.05, ***P < 0.005, *t*-test). (**B**) Schematic representation of CRAG SIMs and SIM mutants used in this study. Three potential SIMs are illustrated in red boxes. *Arrows* indicate mutated amino acids in SIM. *GR*, Glycine rich domain; *NLS*, nuclear localization signal. Filled black indicates CRAG-specific C-terminal domain. (**C**) CRAG SIM mutants failed to activate SRF. Neuro2A cells were transfected with both pSRF-Luc and pRL-CMV with indicated vector. Luciferase activities were assessed 24 hours after the transfection. (*n* = 3; *P < 0.05, ***P < 0.005, *t*-test). (**D,E**) GFP-CRAG formed nuclear inclusions via SIMs. Neuro2A cells transfected with indicated vector were stained with Hoechst 33258 (blue) 24 hours after the transfection. Scale bar, 10 μm. (E) The percentage of cells showing nuclear inclusions of CRAG and SIM mutants. (*n = *3 independent experiments, quantifying at least 50 cells from three coverslips within each experiment; ***P < 0.005, *t*-test).

CRAG interacts with PML bodies^1^, which are also involved in SRF activation^11^. Therefore, we determined whether PML is required for CRAG-induced SRF activation. PML knockdown significantly inhibited CRAG-induced SRF activation (Fig. 4A and Supplementary Fig. 4A). Many SUMOylated proteins and SUMO-binding proteins accumulate in PML bodies^12^. *In silico* analysis using GPS-SUMO software suggested three potential SUMO-interacting motifs (SIMs) in CRAG (Fig. 4B) that mediate non-covalent interactions with SUMO^13^. We examined the effects of these three CRAG SIM mutants (Fig. 4B) on SRF activation. Two CRAG SIM mutants—M1 and M2—did not activate SRF (Fig. 4C), suggesting that these mutants might not associate with PML bodies. To test this possibility, we compared the subcellular distribution of GFP-CRAG SIM mutants (Fig. 4D), because GFP-CRAG forms large nuclear inclusions without stimulation^1^. Consistent with our previous observations, GFP-CRAG and GFP-CRAG SIM-M3 formed large ring nuclear inclusions, whereas GFP-CRAG SIM-M1 and SIM-M2 failed to form nuclear inclusions with PML (Fig. 4E), indicating that SIMs in CRAG are required for the formation of CRAG nuclear inclusions and interaction with PML bodies. Given that overexpression of SUMO1 stabilizes PML bodies^14,15^, we examined the subcellular distribution of GFP-SUMO1 (Supplementary Fig. S4B,C). WT CRAG induced large GFP-SUMO1 nuclear inclusions, whereas CRAG SIM-M1 and SIM-M2 did not. These results collectively suggest that CRAG SIMs play a critical role in recognition of PML bodies and SRF activation.

### CRAG induces ELK1 translocation to PML bodies under MG132 treatment

**Figure 5:**
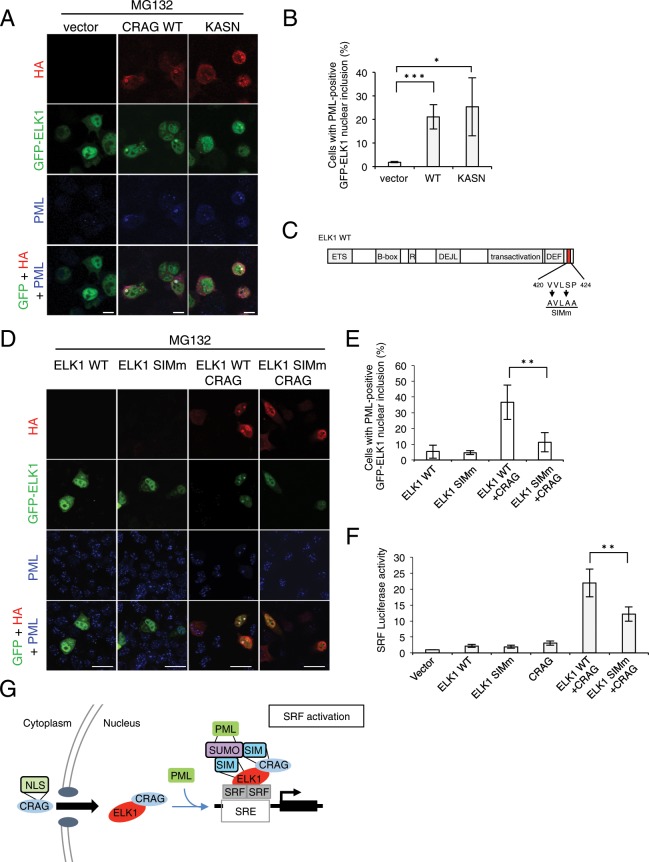
CRAG induces ELK1 translocation to PML body via ELK1 SIM domain. (**A,B**) CRAG induces ELK1 translocation to PML body in a GTPase-independent manner. Neuro2A cells transfected with indicated vector were stained with indicated antibody 24 hours after the transfection. Neuro2A cells were treated with 10 μM MG132 for 4 hours. Scale bar, 10 μm. (**B**) The percentage of cells showing PML-positive GFP-ELK1 nuclear inclusions. (*n = *3 independent experiments, quantifying at least 50 cells from three coverslips within each experiment; *P < 0.05, ***P < 0.005, *t*-test). (**C**) Schematic representation of ELK1 SIM and SIM mutant used in this study. This potential SIM are illustrated in red boxes. *Arrows* indicate mutated amino acids in SIM. (**D,E**) ELK1 SIM is required for ELK1 translocation to PML body. Neuro2A cells transfected with indicated vector were stained with indicated antibody 24 hours after the transfection. Scale bar, 10 μm. (**E**) The percentage of cells showing PML-positive GFP-ELK1 nuclear inclusions. (*n = *3 independent experiments, quantifying at least 50 cells from three coverslips within each experiment; *P < 0.05, *t*-test). (**F**) ELK1 SIM is required for CRAG-induced SRF activation. Neuro2A cells were transfected with both pSRF-Luc and pRL-CMV with indicated vector. Luciferase activities were assessed 24 hours after the transfection. (*n* = 3; **P < 0.01, *t*-test). (**G**) A schematic model for the CRAG-mediated ELK1-SRF activation via PML body. Activated CRAG translocates to the nucleus in an NLS-dependent manner. In the nucleus, CRAG interacts with ELK1 and accumulates at PML body via CRAG SIMs. CRAG and ELK1 cooperatively activate SRF at PML body.

Next, we examined whether CRAG induces ELK1 translocation to PML bodies. Distinct from GFP-CRAG, CRAG requires oxidative stress or proteasome inhibition to form nuclear inclusions co-localised with PML bodies (Supplementary Fig. S5A,B). GFP-ELK1 mainly localized in the nucleus under resting conditions, consistent with a previous study (Supplementary Fig. S5C)^16^. As expected, CRAG induced ELK1 translocation to PML bodies under MG132 treatment (Fig. 5A,B). CRAG KASN also induced ELK1 accumulation at PML bodies, suggesting that the CRAG GTPase domain is not required for ELK1 translocation to PML bodies.

Given that ELK1 also possesses a SIM domain in its C-terminal region (Fig. 5C), we examined whether the SIM domain of ELK1 is involved in CRAG-mediated ELK1 translocation to PML bodies. As expected, mutations in ELK1 SIM significantly attenuated CRAG-mediated ELK1 translocation to PML bodies (Fig. 5D,E), indicating that CRAG induces ELK1 translocation to PML bodies via the ELK1 SIM domain. Consistent with this result, mutations in ELK1 SIM significantly attenuated CRAG-mediated SRF activation (Fig. 5F). Thus, these results indicate that CRAG activates SRF at least in part through ELK1 translocation to PML bodies. A schematic model of CRAG-mediated SRF activation is shown in Fig. 5G.

## Methods

### Mice

CRAG flox/flox mice (Accession No. CDB0630K: http://www2.clst.riken.jp/arg/mutant%20mice%20list.html) were crossed with CAG-Cre mice^17^. The CRAG KO mice were backcrossed for at least 10 generations onto C57B/6J mice. Genotype was confirmed by tail tipping mice at around 1month. Mice were genotyped for CRAG gene using PCR primer A, 5′-CTCAGGATGACTCCCGAACTCTATACGG-3′, and primer B, 5′-CTGGCAGGGCCTGGTAGATGTGCTTCATTG-3′, primer C, 5′-GATTGGCGGCACTGATGGCATCTGTTGG-3′. Southern blot analysis of XmnI and EcoRV digests by using a probe. RT-PCR was performed using primers 5′- GACAGGGACCTATGTCCAGGAAGAGTC-3′ and 5′- GTTTGCGGGCCCTGCTGTCATCAATGACCC-3′. All animals were maintained under university guidelines for the care and use of animals. The experiments were performed after securing Tokyo University of Pharmacy and Life Sciences Animal Use Committee Protocol approval. In c-Fos induction experiments, mice received intraperitoneal injections of either phosphate-buffered saline (PBS) or kainic acid. Kainic acid was from Enzo Life Science.

### Histology and immunohistochemistry

Brains were fixed in 4% PFA, and 40 μm slice sections were prepared. Slices were incubated for 2 nights at 4 °C with primary antibodies. Slices were incubated overnight with Alexa-conjugated secondary antibodies and counterstained with Hoechst 33258. The samples were analyzed using an Olympus FV1000-D confocal fluorescence microscope.

### Cell culture, transfection, and luciferase assay

Mouse primary hippocampal neurons were prepared from embryonic day 18.5 and cultured in Neurobasal medium supplemented with B27. Neuro2A cells were maintained in Dulbecco’s modified Eagle’s medium (DMEM) supplemented with 10% fetal bovine serum (FBS) and penicillin/streptomycin at 37 °C, in 5% CO_2_, in a humidified chamber. Neuro2a cells were transfected with Lipofectamine 2000 or Lipofectamine LTX (Invitrogen) according to the manufacturer’s instructions. Luciferase assay was performed using dual-luciferase reporter assay system (Promega). U0126 was from Cell Signaling. MG132 was from PEPTIDE INSTITUTE, INC.

### Antibodies

Anti-CRAG rabbit polyclonal antibodies were described previously^1^. Anti-α-tubulin, anti-β-actin and anti-FLAG antibodies were from Sigma. Anti-HA antibody was from Babco. Anti-c-Fos, anti-ELK1, anti-Ubiquitin and anti-SRF antibodies were from Santa Cruz Biotechnology.

### Immunofluorescence microscopy

Cells were fixed with 4% PFA in PBS for 15 min at 37°C, then washed twice with PBS, permeabilized with 0.1% Triton X-100 in PBS for 10 min, washed four times with PBS, and blocked with 3% bovine serum albumin in PBS, all at room temperature. For double staining, the cells were incubated with appropriate primary antibodies for 1 h at room temperature, washed three times with PBS, and then incubated with appropriate secondary antibodies for 30 min. The samples were washed as before, mounted using Fluorescent Mounting Medium (Dako), and analyzed using an Olympus FV1000-D confocal fluorescence microscope.

### Whole brain lysates, total cell lysates, co-immunoprecipitation, and western blotting

Mice were anesthetized with isoflurane and decapitated. The brain was immediately removed and washed with ice-cold PBS. The brain was cut into small pieces using a blade and then the brain was homogenized with STM buffer (250 mM sucrose, 50 mM Tris-HCl, pH 7.4, 5 mM MgCl_2_) containing protease inhibitor. The protein concentration was estimated by using The Bio-Rad protein assay (Bio-Rad). Whole brain lysates were separated by SDS-PAGE and transferred to the PVDF membrane (Millipore). The blots were probed with the indicated antibodies, and protein bands on the blot were visualized by the enhanced chemiluminescence reagent (Millipore). To immunoprecipitation assay, the brain was homogenized with 6 times the amount of lysis buffer (20 mM Tris-HCl, pH 7.4, 5 mM EDTA, 1% Triton X-100, 0.1% SDS, 150 mM NaCl) containing protease inhibitor. The lysates were sonicated and were clarified by centrifugation at 15,000 X g for 10 min and immunoprecipitated with the appropriate antibody. Immunoprecipitates were washed three times with lysis buffer. Samples were then subjected to SDS-PAGE and Western Blot.

Cells were rinsed twice with PBS and harvested and lysed directly using 1 × sample buffer (37.5 mM Tris-HCl, pH 6.8, 9% Glycerol, 9% SDS, 0.01% bromophenol blue, 2.5% 2-mercaptoethanol). Total cell lysates were sonicated and equal amounts of whole cell lysates were subjected to SDS-PAGE and Western Blot.

Cells were lysed in lysis buffer (20 mM Tris-HCl, pH 7.4, 5 mM EDTA, 1% Triton X-100, 0.1% SDS, 150 mM NaCl). The lysates were sonicated and were clarified by centrifugation at 15,000 × *g* for 10 min and immunoprecipitated with the appropriate antibody. Immunoprecipitates were washed three times with lysis buffer. Samples were then subjected to SDS-PAGE and Western Blot.

### GTP-binding assays

Cells transfected with indicated plasmids were solubilized in lysis buffer (20 mM Tris-HCl, pH 7.4, 5 mM EDTA, 1% Triton X-100, 0.1% SDS, 150 mM NaCl) containing protease inhibitor. The lysates were sonicated and were clarified by centrifugation at 15,000 × *g* for 10 min and the supernatant was incubated with 30 μl GTP-agarose beads (Sigma) on ice for 60 minutes, or at 30°C, for the indicated times. The resin was washed three times with lysis buffer. The proteins were resolved by SDS-PAGE and analyzed by immunoblotting.

### Expression constructs

CRAG WT, S140N mutant, NLS mutant, ΔC mutant, ΔN mutant, centaurin-γ3/AGAP3, Myc-SRF, and SRF mutantsΔ338 were described previously^4^. ELK1, ELK3 and ELK4 were obtained from mouse brain by RT-PCR. These cDNA with N-terminal FLAG epitope tag were created by PCR using the ELK1 primer F, 5′-CCGAATTCCATGGACCCATCTGTGACGCTG-3′, ELK1 primer R, 5′-CTGGATCCTTTCATGGCTTCTGGGGCCCTG-3′, ELK3 primer F, 5′-CCAAGCTTATGGAGAGTGCAATCACGCTG-3′, ELK3 primer R, 5′-GGTCTAGATTAGGATTTCTGAGAGCTGGG-3′, ELK4 primer F, 5′-CCGAATTCATGGACAGTGCCATCACTCTG-3′, ELK4 primer R and subcloned into pCMV5. GFP-ELK1 was subcloned into pEGFP-C1. Lentivirus mediated CRAG was subcloned into pFUW. cDNA containing the CRAG mutations K139A/S140N (KASN), V129A/V132A(SIM-M1), L174A/I177A(SIM-M2), V226A/V229A(SIM-M3), I92A/L95A/L97A(NESm) were generated with the Site-Directed Mutagenesis Kit (Stratagene). cDNA containing the ELK1 mutations M55K, M55K/S384A/S390A (ELK1-SA), or M55K/S384D/S390D (ELK1-SD) were generated with the Site-Directed Mutagenesis Kit. We used ELK1 M55K (ELK1 WT) not to generate short isoform of ELK1. cDNA containing CRAGΔGTP, centaurin-γ3 ΔGTP, CRAG GTPase mutants and ELK1ΔETS were amplified from the full-length cDNA by PCR. CRAG-NLS, CRAG KASN-NLS were generated by addition of NLS (KKKRKV) to the full-length cDNA by PCR. pAP-1-Luc plasmid and pSRF-Luc plasmid were from Stratagene. pRL-CMV was used as an internal control reporter from Promega. *c-fos*-promoter-Luc was obtained from the total genome of mouse brain by PCR using the primers 5′-CCACGCGTATGTTCGCTCGCCTTCTCT-3′ and 5′-GGAGATCTTGCTCGTTCGCGGAACC-3′ and subcloned into pGL3-basic vector. For RNAi assay, sense and antisense oligonucleotides corresponding to the following target sequences were designed: 5′-AGTTGGTGGATGCAGAGGA-3′ (siELK1-a) and 5′-CAGCCTGAGGTGTCTGTAA-3′ (siELK1-b). siPML-a(SI01382514) and siPML-b (SI02734347) were from Qiagen. Qiagen’s thoroughly tested and validated AllStars Negative Control siRNA was used as a negative control.

## Discussion

We previously demonstrated that CRAG induces SRF-mediated c-Fos activation in cultured neuronal cells^4^, but the physiological relevance of CRAG *in vivo* was heretofore unknown. In the present study, kainic acid-induced SRF activation was severely attenuated in the hippocampal neurons of WKO mice. Thus, CRAG might regulate neuronal development via SRF-mediated gene expression. However, SRF activation was not completely blocked by CRAG deletion, suggesting that CRAG is involved in one of the SRF-activating pathways. Compared to ubiquitous expression of SRF, CRAG appears to be enriched in brain. In fact, the phenotypes of WKO mice differ from those of SRF-KO mice in some respects: Central nervous system (CNS)-specific SRF-KO mice exhibit a more severe phenotype than WKO mice because they die at the neonatal stage^18^. CRAG might play an important role for the brain development after birth because CRAG expression increases after birth^5^. The different expression patterns of these proteins might make differences in the phenotypes between CRAG WKO and SRF-KO mice.

We also gained insights into the molecular mechanisms of SRF activation by CRAG. We found that CRAG specifically interacts with ELK1, a cofactor of SRF; this finding is concordant with previous results showing that found CRAG induces c-Fos, which is one of the immediate early genes. Since ELK1 shuttles between nucleus and cytoplasm^16^, CRAG can interact with ELK1 inside or outside the nucleus. CRAG might be activated by various physiological stimulations that cause oxidative stress and subsequently induce SRF and c-Fos activation via ELK1.

Our study also demonstrated the regulatory mechanism of CRAG. We found that the CRAG GTPase domain is essential for SRF activation (Fig. 3). Although the novel GTPase mutant KASN localized at the nucleus, it did not activate SRF. Because GTPase domains regulate protein–protein interactions, the CRAG GTPase domain might be crucial for forming a complex to activate SRF. Further investigation is needed to identify target protein(s) activated by the CRAG GTP-bound form. It is also important to understand how CRAG GTPase is activated in redox signalling and oxidative stress. We previously observed that endogenous CRAG is mainly localized in cytosol and forms nuclear inclusions in response to oxidative stress^1^. The distribution of GFP-CRAG is different from that of HA-CRAG and endogenous CRAG. GFP-CRAG accumulates in the nuclear inclusions associated with PML bodies without stimulation. We consider that GFP-tagging at the N-terminal of CRAG caused a conformational change of CRAG and induced active form of CRAG. This suggests an unknown modification-mediated CRAG activation and subsequent nuclear translocation. In the present study, we found that CRAG was phosphorylated at multiple sites, suggesting that CRAG GTPase is regulated by phosphorylation. However, the kinase that phosphorylates CRAG remains unknown. We are currently screening for this kinase by using the kinase inhibitor library.

PML bodies act as a signalling platform for CRAG to activate SRF cooperatively with ELK1. SRF has been shown to be modified by SUMO1^19^ and regulated by PML^11^. PML is SUMOylated at three different sites and contains a SIM^15^. In addition, the SIM in PML-binding proteins is required for PML body localization. These findings are consistent with our observation that CRAG SIM is crucial for forming nuclear inclusions co-localised with PML. ELK1 also possesses a SIM domain in its C-terminal region, and CRAG induces ELK1 translocation to PML bodies through the ELK1 SIM domain (Fig. 5C). Thus, SUMOylation and SUMO–SIM interactions constitute a regulatory mechanism of CRAG-mediated SRF activation via ELK1. Nevertheless, further investigation is needed to identify the roles of CRAG-mediated SRF activation in neuronal development and the pathogenesis of neurodegenerative diseases.

## Supplementary information

Supplementary Information

## Data Availability

The data in the current study are available from the corresponding author on reasonable request.
