# Supplementary material for: Critical role of CRAG, a splicing variant of centaurin-γ3/AGAP3, in ELK1-dependent SRF activation at PML bodies
Source: Sci Rep. 2019 Dec 27;9:20107. doi: 10.1038/s41598-019-56559-9 (PMC6934726; doi:10.1038/s41598-019-56559-9)
Supplement: Supplementary file 1 — Supplementary Information [file 41598_2019_56559_MOESM1_ESM.pdf]

Supplementary information

Critical role of CRAG, a splicing variant of centaurin-γ3/AGAP3, in ELK1-dependent SRF activation at PML bodies

Shun Nagashima[1], Keisuke Takeda[1], Isshin Shiiba[1], Mizuho Higashi[1], Toshifumi Fukuda[1], Takeshi Tokuyama[1], Nobuko Matsushita[1], Seiichi Nagano[2], Toshiyuki Araki[2], Mari Kaneko[3,4], Go Shioi[4], Ryoko Inatome[1] and Shigeru Yanagi[1]*

[1]Laboratory of Molecular Biochemistry, School of Life Sciences, Tokyo University of Pharmacy and Life Sciences, Hachioji, Tokyo 192-0392, Japan

[2]Department of Peripheral Nervous System Research National Institute of Neuroscience, National Center of Neurology and Psychiatry, Kodaira, Tokyo, Japan

[3]Animal Resource Development Unit and [4]Genetic Engineering Team, Division of Bio-function Dynamics Imaging, RIKEN Center for Life Science Technologies, Kobe, Japan

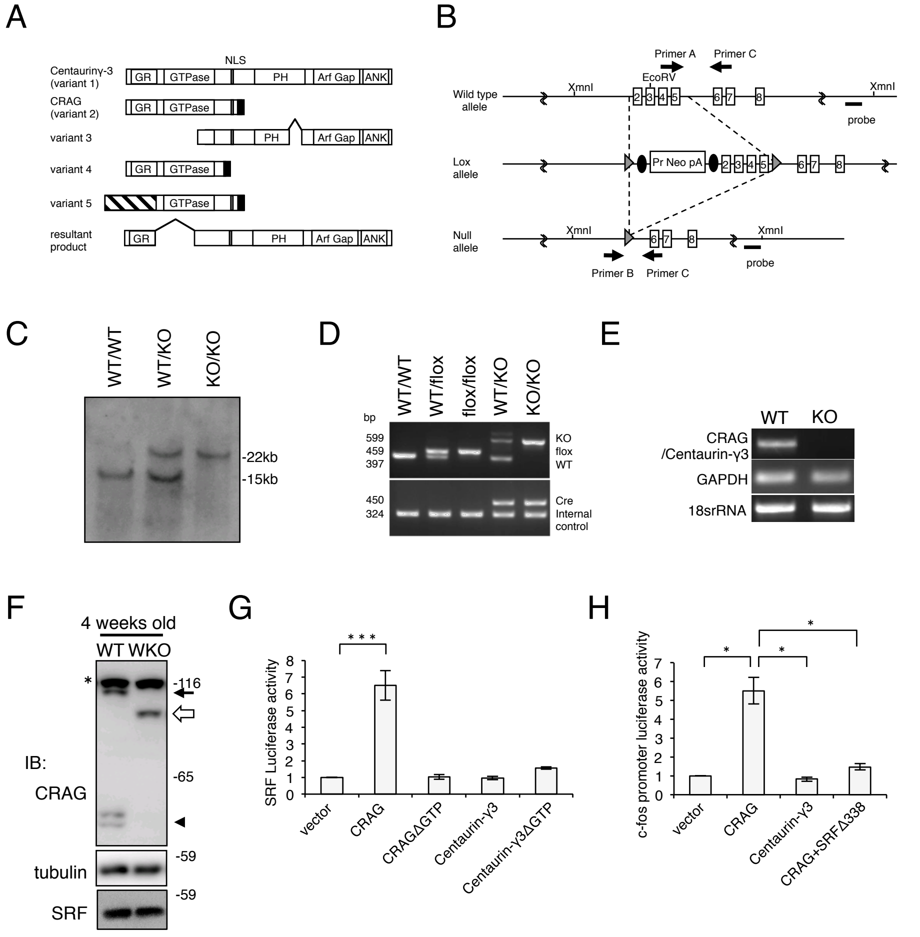

Supplemental Figure 1.

Generation of CRAG/centaurin-γ3 KO mice.

(A) Structural comparison of CRAG with centaurin-γ3/AGAP3 and GTPase domain-deleted variant (variant 3). *GR*, Glycine rich domain; *NLS*, nuclear localization signal; *ANK*, ankyrin repeat; *PH*, pleckstrin homology domain. Filled black indicates CRAG-specific C-terminal. (B) A schematic diagram of the genomic mouse *CRAG* gene. The genomic structure of the wild-type *CRAG* allele (top), the lox allele (middle) and the null allele (bottom) are shown. Targeting strategy for *CRAG* gene: homologous recombination of the targeting vector and generation of the *CRAG* knockout by activation of the Cre-loxP system. Triangles, loxP sites; Neo, neomycin resistance gene; filled circles, frt site; XmnI, XmnI restriction sites; EcoRV, EcoRV restriction sites; horizontal bar, probe (used for southern blot analysis); allows, PCR primers (used for genome PCR). (C) Southern blot analysis with the probe to confirm activation of the Cre-loxP system and generation of *CRAG* knockout at P18. The probe hybridized with 15kb and 22kb XmnI and EcoRV fragments from the WT or knocked out alleles, respectively. (D) PCR genotyping. WT, heterozygous and homozygous mice were identified by the amplification of PCR products specific for either the *CRAG* wild-type allele (397bp), the lox allele (459bp) or the null allele (599bp). The presence of the *Cre* gene was identified by the amplification of PCR products specific for *Cre* (450bp) or *GAPDH* (324bp) as internal control. (E) RT-PCR analysis to confirm loss of *CRAG* mRNA. *GAPDH* and *18srRNA* were internal controls. (F) Western blot analysis demonstrating loss of CRAG and centaurin-γ3 expression in whole brain of whole-body KO (WKO) at 4 weeks old. Arrow heads, CRAG; filled arrow, centaurin-γ3; unfilled arrow, centaurin-γ3 ΔGTPase; Asterisk indicates non-specific band. (G) Centaurin-γ3 ΔGTP does not activate SRF. Neuro2A cells were transfected with both pSRF-Luc and pRL-CMV together with indicated vector. Luciferase activities were assessed 48 hours after the transfection. (n = 3; ***$P < 0.05$, t-test). Error bars indicate S.D. (H) CRAG activates c-fos promoter via SRF. Neuro2A cells were transfected with both *c-fos* promoter-Luc and pRL-CMV together with an either empty expression vector or an indicated vector. SRF Δ338 mutants exert a dominant negative effect. Luciferase activities were assessed 48 hours after the transfection. (n = 3; *$P < 0.05$, t-test). Error bars indicate S.D.

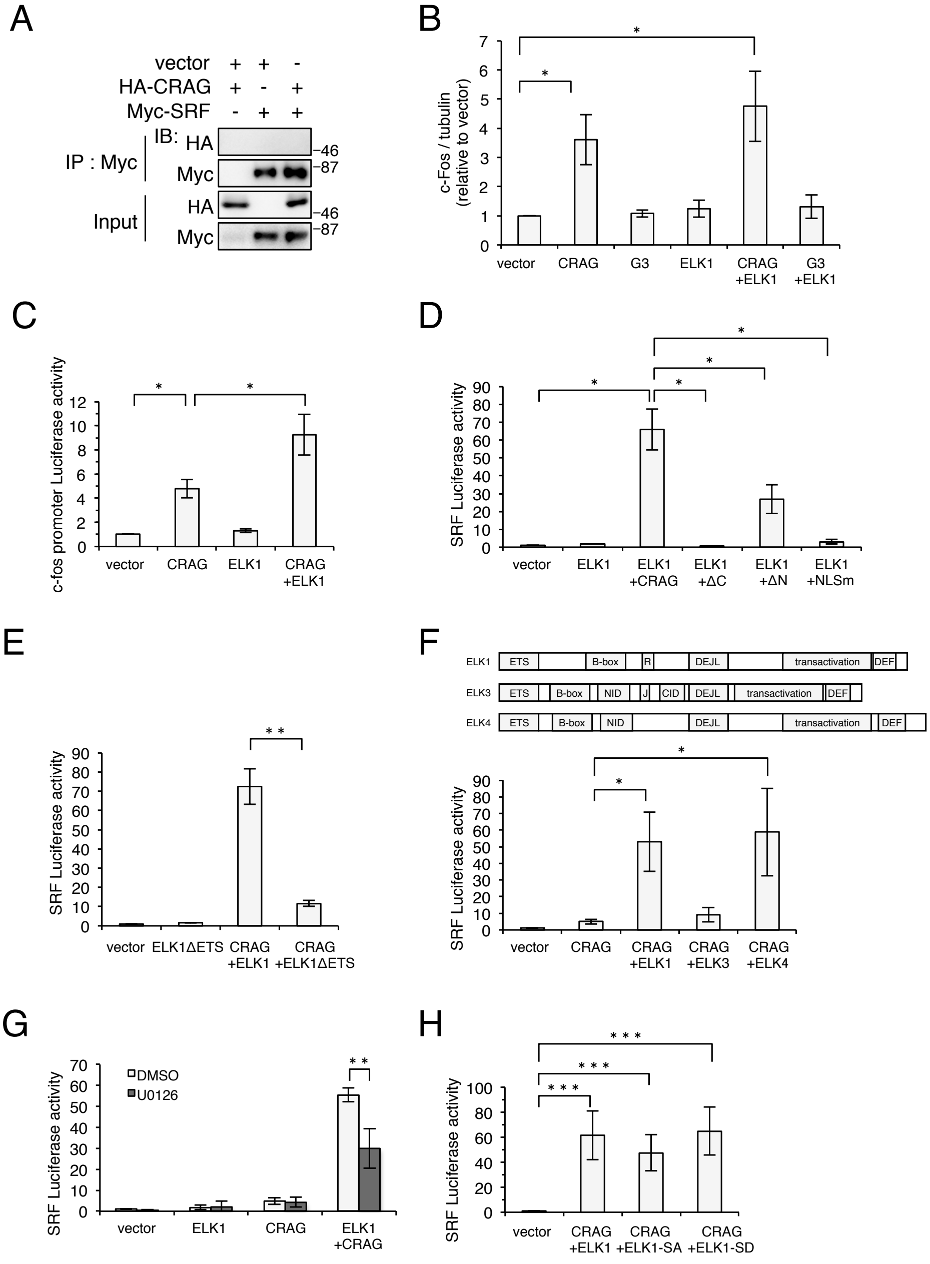

Supplemental Figure 2.

Cooperative activation of SRF by CRAG and ELK1.

(A) CRAG dose not interact with SRF. Lysates of Neuro2a cells transfected with the indicated vectors were sonicated and subjected to an IP-IB assay with the indicated antibodies. These blots of HA and Myc were obtained from different exposure times between IP: myc and Input depending on signal intensities.

(B) Quantification of the expression levels of c-Fos in Fig. 2E. (n = 3; *P < 0.05, t-test).

(C) Synergistic activation of c-fos promoter by CRAG and ELK1. Neuro2A cells were transfected with both c-fos promoter-Luc and pRL-CMV together with either empty expression vector or indicated vector. Luciferase activities were assessed 48 hours after the transfection. (n = 3; *P < 0.05, t-test).

(D) Effects of various CRAG mutants on cooperative activation of SRF with ELK1.

(E) ETS domain of ELK1 is essential for CRAG-induced SRF activation.

(F) Effects of other ELK family members on CRAG-induced SRF activation.

(G) MEK inhibitor partially inhibits SRF activation by CRAG and ELK1.

(H) Effects of ELK1 phosphorylated (ELK1-SD) and non-phosphorylated (ELK1-SA) mutants on CRAG-induced SRF activation.

(D-H) Luciferase assay was performed with Neuro2A cells transfected with both pSRF-Luc and pRL-CMV with indicated vector. (n = 3; *P < 0.05, **P < 0.01, ***P < 0.005, t-test). All error bars indicate S.D.

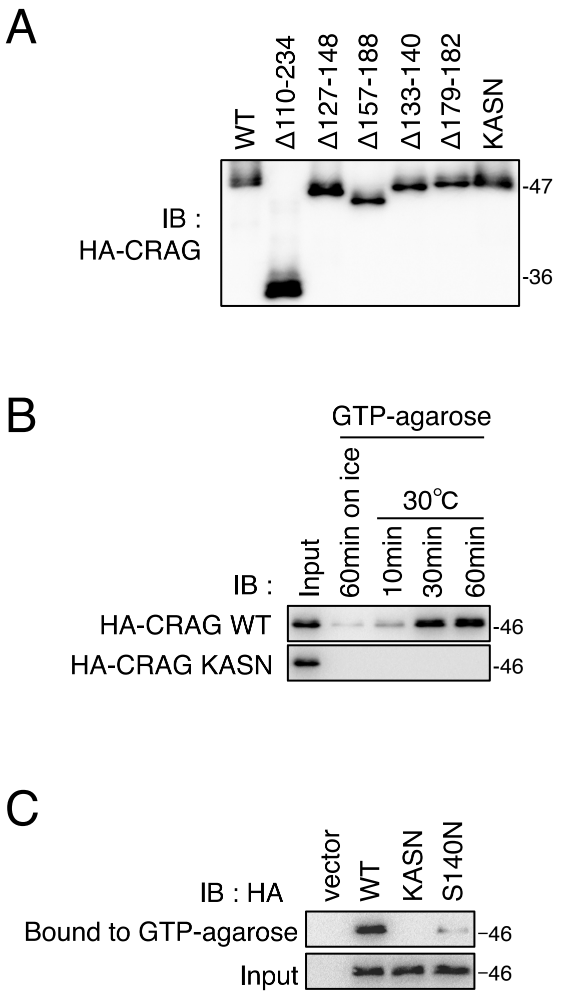

Supplemental Figure 3.

Characterization of various CRAG GTPase mutants

(A) Expressions of CRAG-WT and various CRAG GTPase mutants. Neuro2A cells were transfected with indicated constructs. Lysates of Neuro2A cells were immunoblotted with anti-HA.

(B and C) A CRAG KASN mutant failed to bind GTP.

(B) HA-tagged CRAG-WT and KASN expressed in Neuro2a cells were solubilized by lysis buffer. The lysates were incubated with GTP-agarose beads on ice for 60 minutes, or at 30°C, for the indicated times. The bound proteins were analyzed by immunoblotting using anti-HA antibodies.

(C) HA-tagged CRAG-WT, KASN and S140N mutants were incubated with GTP-agarose at 30°C for 1 hour and bound proteins were analyzed by immunoblotting using anti-HA antibodies.

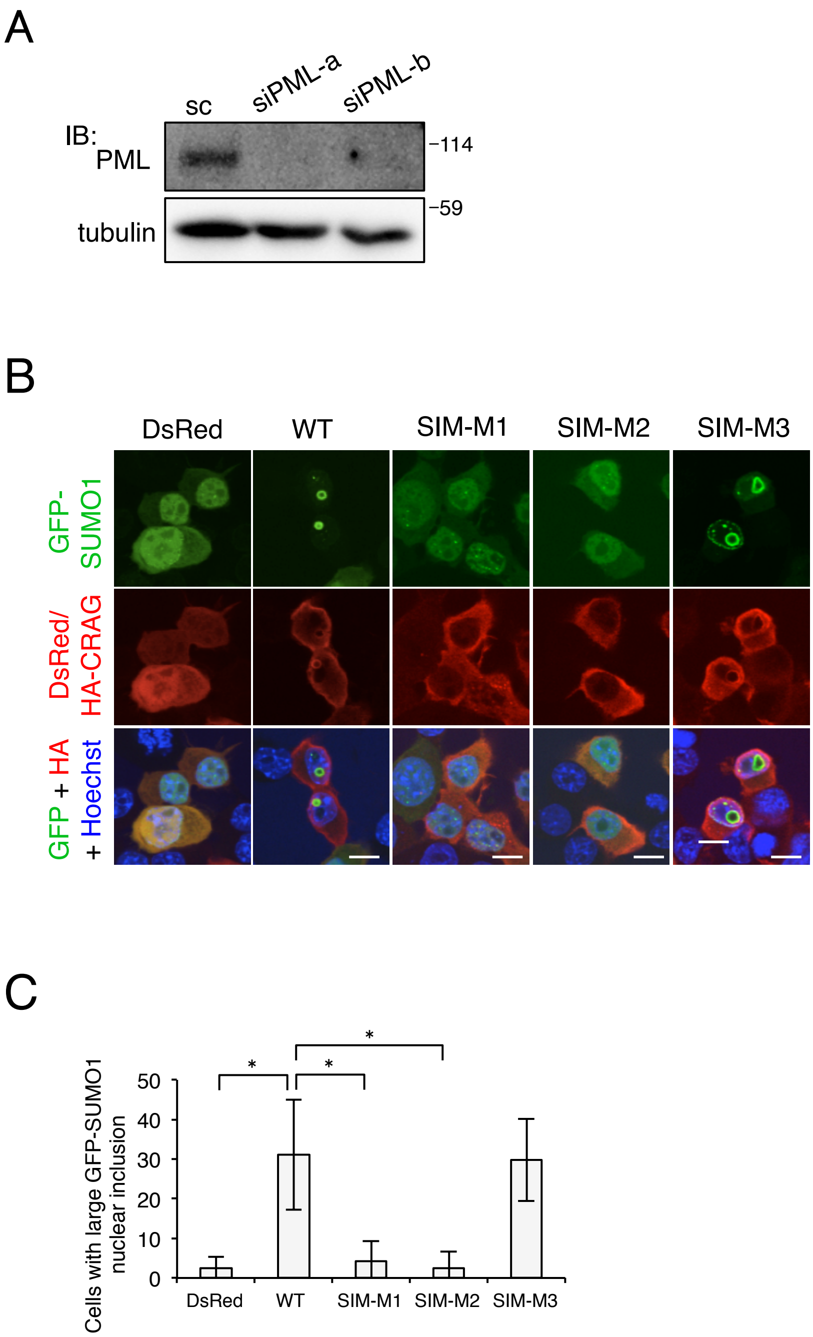

Supplemental Figure 4.

Characterization of various CRAG SIM mutants

(A) Western blot analysis to confirm PML knockdown. Neuro2A cells were transfected with either the scramble siRNA (sc) or the PML siRNA for 48 h. (B and C) CRAG SIMs were required for CRAG-induced large GFP-SUMO1 nuclear inclusion. Neuro2A cells transfected with either DsRed, HA-CRAG WT, or HA-CRAG SIM mutants and GFP-SUMO1 were immunostained with anti-HA (red) and Hoechst 33258 (blue) 24 hours after the transfection. Scale bars represent 20 µm. The percentage of cells showing large GFP-SUMO1 nuclear inclusion (*n* = 3 independent experiments, quantifying at least 50 cells from three coverslips within each experiment; *P <0.05, *t*-test). All error bars indicate S.D.

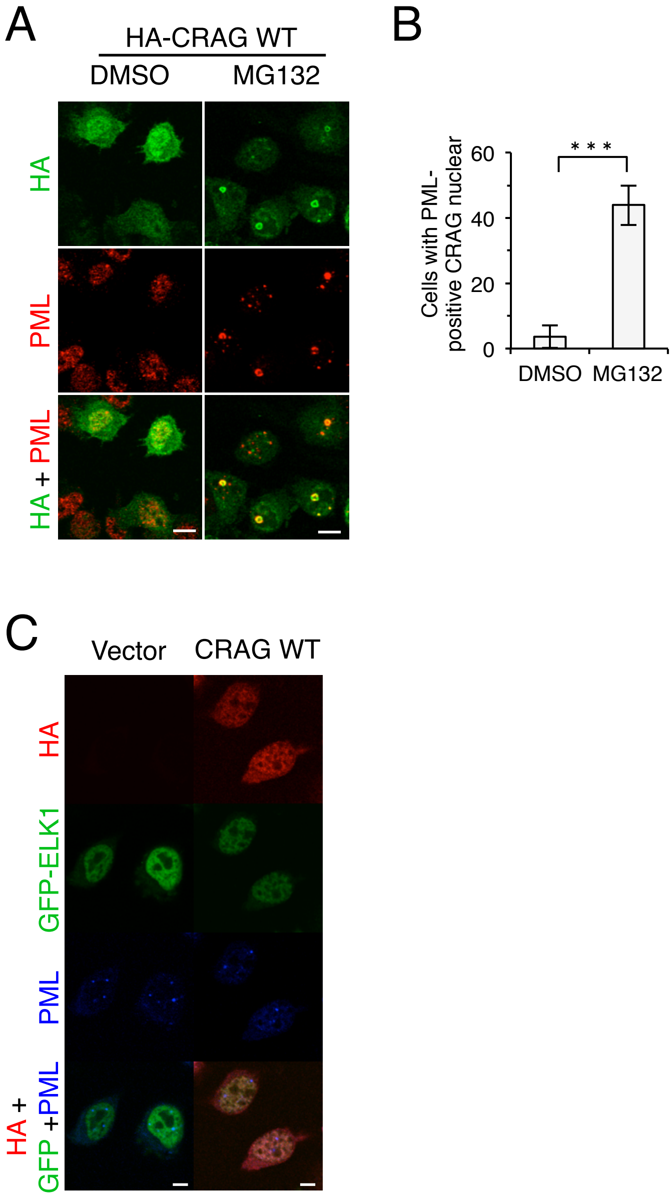

Supplemental Figure 5.

CRAG translocates to PML body under MG132 treatment

(A and B) CRAG accumulation at PML body after MG132 treatment. Neuro2A cells transfected with HA-CRAG WT were immunostained with anti-HA (green) and anti-PML (red). Neuro2A cells were treated with either DMSO or 10 mM MG132 for 4 hours. Scale bar, 10 mm. (B) The percentage of cells showing PML-positive CRAG nuclear inclusions. (n = 3 independent experiments, quantifying at least 50 cells from three coverslips within each experiment; ***$P < 0.005$, t-test).

(C) GFP-ELK1 mainly localized to the nucleus in the presence or absence of CRAG. Neuro2A cells transfected with indicated vector were immunostained with anti-HA (red) and anti-PML (blue). Scale bar, 10 mm.

Supplemental figure 6 part1

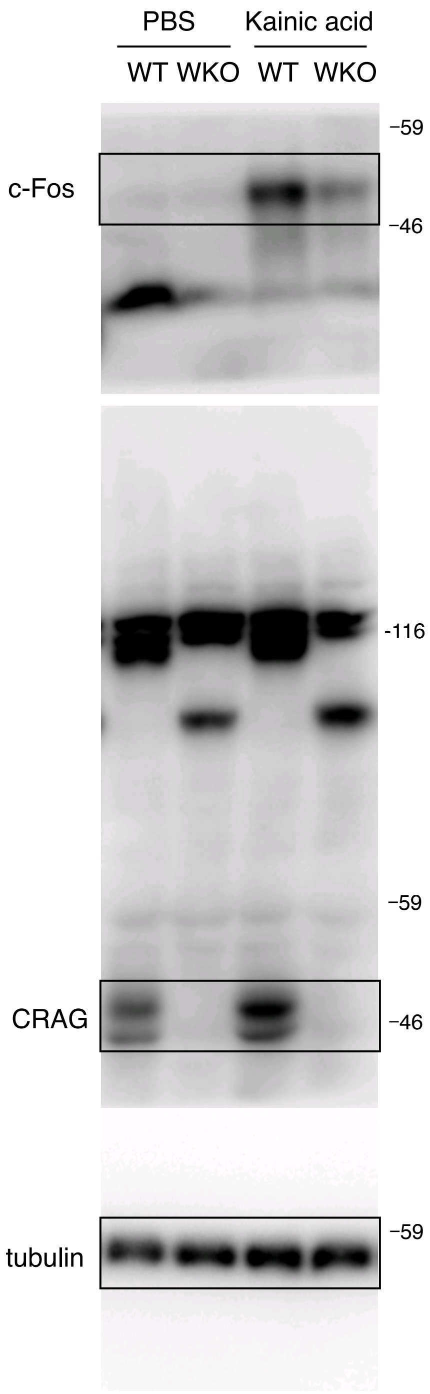

Original scan for Fig. 1B

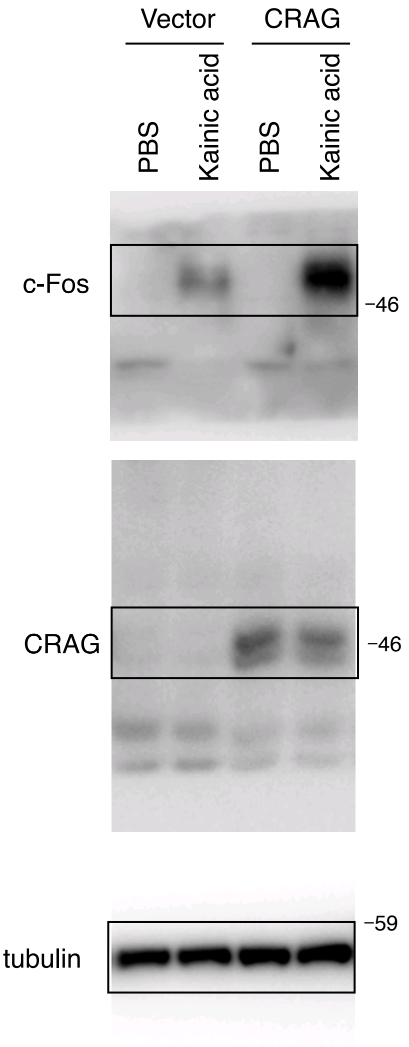

Original scan for Fig. 1C

Supplemental figure 6 part2

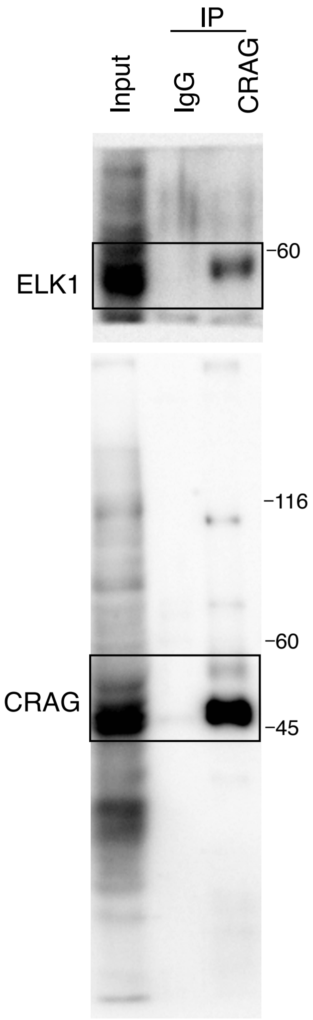

Original scan for Fig. 2A

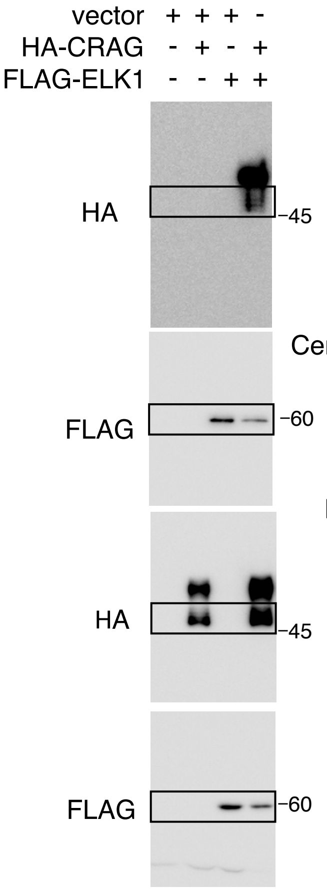

Original scan for Fig. 2B

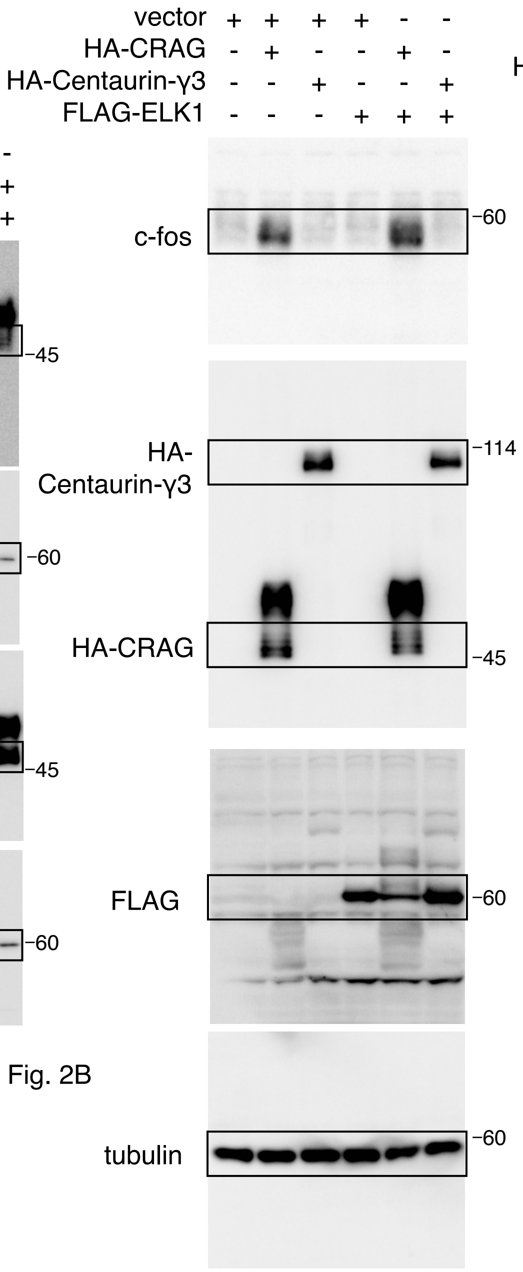

Original scan for Fig. 2E

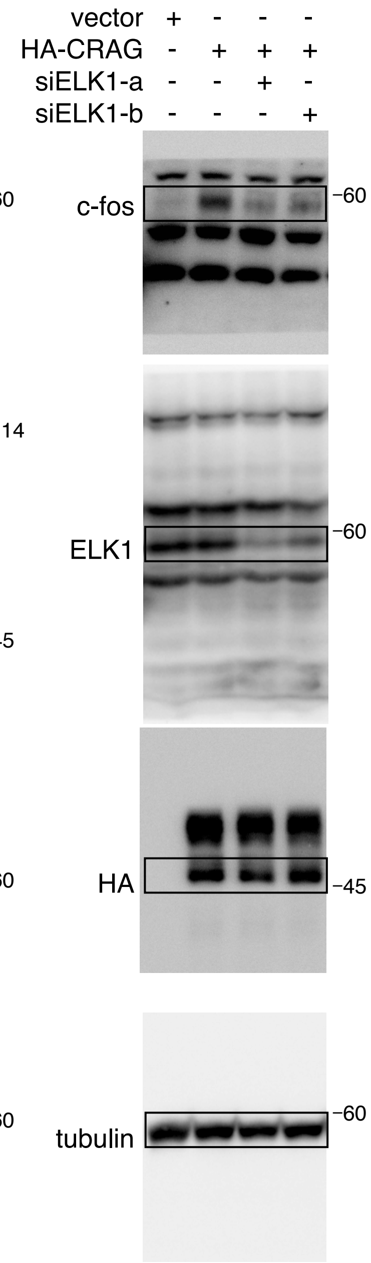

Original scan for Fig. 2F

Supplemental figure 6 part3

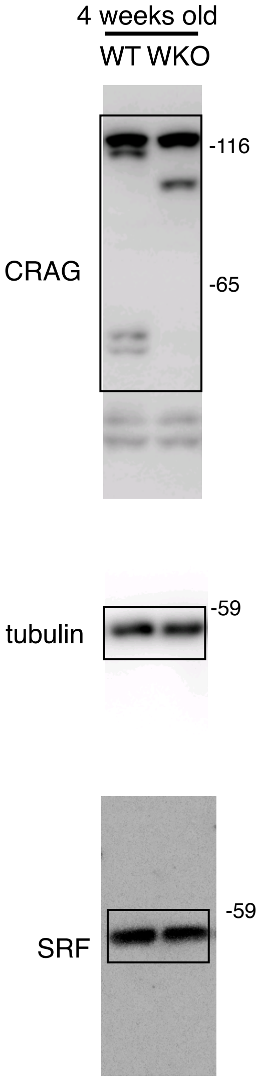

Original scan for Sup Fig. 1F

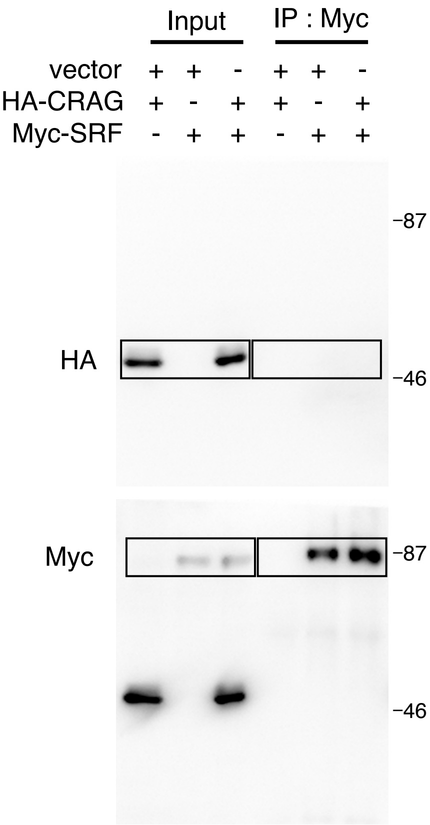

Original scan for Sup Fig. 2A

Supplemental figure 6 part4

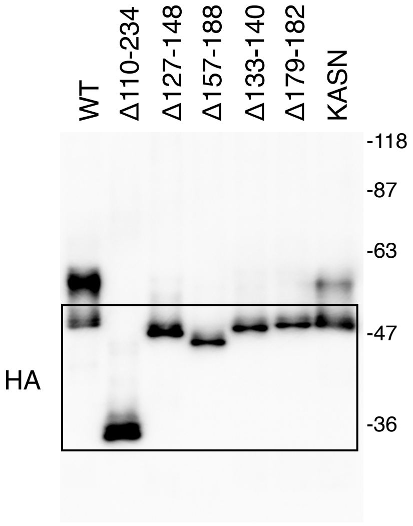

Original scan for Sup Fig. 3A

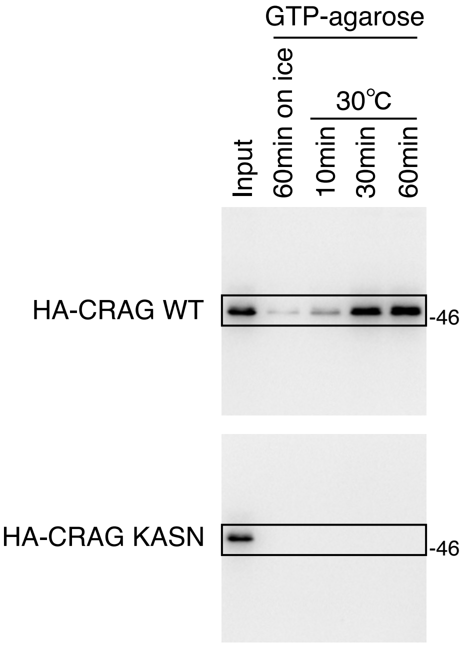

Original scan for Sup Fig. 3B

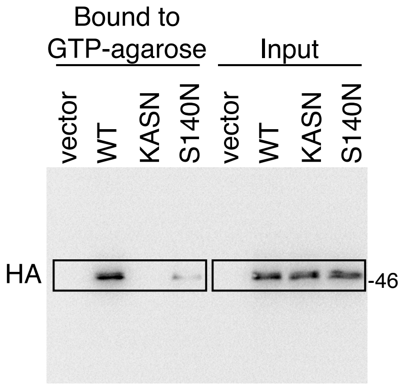

Original scan for Sup Fig. 3C

Supplemental figure 6 part5

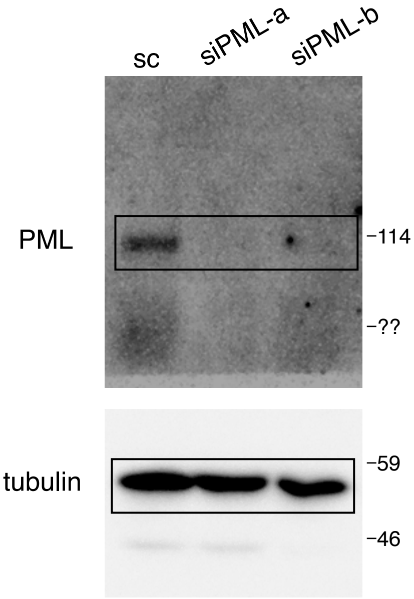

Original scan for Sup Fig. 4A
